# Comparison of the Perspectives of Managers, Employees and Clients Regarding the Individual Barriers of Family Planning Counseling in Healthcare Centers of Isfahan in 2012

**DOI:** 10.5812/ircmj.15075

**Published:** 2014-03-05

**Authors:** Safoura Taheri, Soheila Ehsanpour, Shahnaze Kohan, Saba Farzi, Molouk Jaafarpour, Ashraf Direkvand-Moghaddam

**Affiliations:** 1 Department of Midwifery, School of Nursing and Midwifery, Ilam University of Medical Sciences, Ilam, IR Iran; 2 Department of Medical Education , Medical Education Research Center, Isfahan University of Medical Sciences, Isfahan, IR Iran; 3 Nursing and Midwifery Care Research Center, School of Nursing and Midwifery, Isfahan University of Medical Sciences, Isfahan, IR Iran; 4 Ayatollah Madani Hospital, Lorestan University of Medical Sciences, Lorestan, IR Iran; 5 Research Center for Social and Psychological Injuries, Ilam University of Medical Sciences, Ilam, IR Iran

**Keywords:** Family Planning, Counseling, Contraception, Barrier, Iran

## Abstract

**Background:**

Family planning is a lifestyle that is selected voluntarily and is based on the knowledge, attitude and responsible decision making by couples in order to promote the health and welfare of the family and the advancement of the society. In this regard, family planning counseling plays an important role in making informed decisions if used properly and in a responsible way. Detection of individual barriers in family planning counseling based on the viewpoints of managers, employees and clients who are key participants in the healthcare service provision is a major step towards appropriate planning to modify or eliminate such barriers.

**Objectives:**

The present study was conducted with the goal of comparing managers’, employees’ and clients’ viewpoints about individual barriers in family planning counseling in health care centers in Isfahan in 2012.

**Patients and Methods:**

This was a cross-sectional one-step three-group comparative descriptive study conducted on 295 subjects including 59 managers, 110 employees and 126 clients in medical health care centers in Isfahan in 2012. The managers and employees were selected by census sampling, and the clients were recruited through convenient random sampling. The data collection tool was a researcher-designed questionnaire, which was designed in two sections of fertility and personal characteristics, and viewpoint measurement. Descriptive and inferential statistical tests were used to analyze the data.

**Results:**

The obtained results showed significant differences between mean scores of viewpoints in three groups of managers, employees and clients concerning individual barriers in family planning counseling. In addition, most of the managers, employees and clients reported individual barriers as an intermediate level barrier in the process of family planning counseling.

**Conclusions:**

Results indicate that subjects in three studied groups hold different views regarding the individual barriers in family planning counseling. This difference in the perspectives may be a factor that affects the quality of the provided services. Therefore, it is necessary for the healthcare providers to consider the main concerns of their clients regarding family planning.

## 1. Background

Statistics show that in every minute, 308 women become pregnant around the world, 198 women unplanned, 110 women with complications associated with pregnancy, 40 women with unsafe abortions and one woman dies, which in most of the cases, is preventable (1). Family planning goals include enabling couples and individuals to make informed and responsible decisions regarding the number and children, time space between the pregnancies, and ensuring that an effective and reliable methods for prevention of pregnancy is provided (2). The core of family planning relies on establishing effective communication between the service providers and their clients. Through providing accurate and complete information, a good counseling can help the client in selecting an appropriate method for family planning, and also in efficient and sustainable use of the selcted methods and in adopting the suitable behavior in facing the common side effects of contraceptive methods. Nevertheless, forming a helpful counseling relationship is not easy. This human connection needs socially alert providers in the field of family planning methods with excellent interpersonal skills (3), and also accepting the recommendations of the counselor by the client in order to achieve the goals of counseling (4). Family planning counseling has a key role in the clients' compliance and the positive impact of the family planning methods and is essential for informed and voluntary decision- making. Unfortunately, the quality of family planning counseling can be poor due to several reasons. Previously, some qualitative studies have been performed in the field of family planning, which have discussed several barriers in the process of family planning counseling. Among them, are the individual barriers. In some of the previously conducted qualitative studies regarding the quality of family planning services, one barrier has been determined as the lack of knowledge of the healthcare workers in providing the correct advice from the patients' perspectives (5, 6). Baraitser, Blake and Brown (2003) investigated the barriers to the involvement of clients in family planning service development by interviewing with the clients. They found that the prejudicial attitude of the healthcare providers was a very important barrier for the clients to access quality family planning services (7). Although qualitative research has suggested these barriers, they have not been yet studied through a quantitative research study in Iran. Detection of the most important and efficient barriers, can speed up the initial steps to eliminate or modify these barriers more efficiently, and enhance the quality of family planning counseling. On the other hand, comparison of viewpoints in the three groups of managers, employees and clients can guide us regarding the possible weaknesses in family planning counseling. The dissimilarity of these barriers from the viewpoints of various individuals may lead to conflicts between them and might result in failure in eliminating or controlling these barriers and eventually, a failure of family planning counseling.

## 2. Objectives

Therefore, in order to improve the quality of the current counseling services and to reach the family planning goals, and considering the importance of various viewpoints, the present study aimed to compare managers’, employees’ and clients’ viewpoints about individual barriers in family planning counseling in health care centers of Isfahan, Iran in the year 2012.

## 3. Patients and Methods

The present study was a comparative descriptive cross- sectional one-step three-group study on 295 subjects including 59 managers, 110 employees and 126 clients studied between the July to October of 2012 in Isfahan, Iran. Method for determining the sample size was: N = (Z1+Z2)^2^S^2^/d^2^ = 63, Z1 = 1.96, Z2 = 0.84, d = 0.05s, Therefore, based on the objectives, sample size was estimated to be at least 63 people in each group. Due to some restrictions, the number of managers recruited into the study was lower. Total of 20 health care centers were selected randomly to enroll their employees and clients into the study. Sampling method was the census method for employees and convenient random sampling for clients. Inclusion criteria for clients were the married women aged 15-49 years who were using one or more of the contraception methods. The managers were selected through census sampling from 44 health care centers. The Ethics Committee of the Midwifery and Nursing department of the Isfahan University of Medical Sciences approved this study (at July 23, 2012 with approval No. 391246). The data were collected using a researcher-designed questionnaire which included two sections. The first section contained questions on managers’, employees’ and clients’ personal and fertility variables, and the second section included a viewpoint survey questionnaire of individual barriers in family planning counseling. The viewpoints about individual barriers in family planning were investigated by mean obtained score of answering 11 five-point scale questions in the viewpoint survey questionnaire. The answers were scored as absolutely disagree (Grade 0), disagree (Grade 1), no idea (Grade 2), agree (Grade 3) and absolutely agree (Grade 4). Individual barriers were defined as the barriers such as lack of privacy, inadequate educational equipment and inadequate various supplies of contraceptives in health care centers (Table 2). Content validation was used to confirm the validity of the questionnaire, performed by using textbooks and valid articles and was re-confirmed by ten specialists in the field of family planning. Reliability of the questionnaire was confirmed through Cronbach’s alpha test (Cronbach's alpha coefficient = 0.87). The data were collected through a self-report questionnaire in two groups of managers and employees, and self-report or questioning in the clients group. It should be noted that four persons in the managers group, six in the staff group and five in the clients group were not willing to participate in the study, for reasons such as lack of time to answer the questions. The data were analyzed by descriptive and inferential statistical methods (One way ANOVA) using SPSS 19 software.

## 4. Results

Personal and fertility characteristics of the managers: there were 59 subjects (33 females and 26 males) with mean age of 43 ± 5.5 years, with an official managerial experience of 10±6 years, of whom 79.7% were general physicians, and 54.2% had two children. In the employees' group, there were 110 subjects with mean age of 39.7 ± 6.2 years with 13 ± 6.7 years work experience in family planning counseling; of these group, 54.6% had B.S. degree in midwifery and 50.9% had two children. Regarding the employees’ responses, mean daily number of clients referring to the center to receive general health care and family planning counseling was calculated to be 17 ± 8 and 10 ± 6, respectively. The majority of them reported to have received updated educational/advertising brochures about family planning counseling (74.5%) and have improved their communication skills through training courses in family planning counseling (82.7%).

Personal and fertility characteristics of the clients: there were 126 subjects with mean age of 29.7 ± 5.8 years, 88.1% were repetitive referrals; 46.8% of subjects had lower than diploma level education, 92.1% were housewives and 56.1% had only one child. The mean number of children that they preferred to have was 2.3. In the majority of clients, condoms were the current method of contraception (42.9%) and previously used method was natural contraception (40.5%). Most of them were satisfied with the current contraceptive method (49.2%) and were partially satisfied with family planning counseling provided in the healthcare centers (50.8%) and their experience of unintended pregnancies (24.6%); their desire for future pregnancies was reported to be relatively high (57.1%).

The mean score for the perspectives regarding the individual barriers was: in the managers group: 53.30 ± 14; in the employees: 49.40 ± 16.75 and in the clients group as 42.5 ± 14. Thus, the highest and lowest mean score was related to the managers and clients groups, respectively. In agreement with the study hypothesis, one-way ANOVA test indicated that the mean score of the perspectives about the individual barriers was significantly different between the three groups (P < 0.001). LSD post hoc test indicated that, there were no significant differences between the managers and employees points of view (P = 0.11). However, there was a significant difference between the scores given by the clients and the score of the managers and employees (P < 0.001). On the other hand, clients considered individual barriers as less influential obstacles in family planning consultation process compared to the other two groups.

Based on the obtained scores from the questions about individual barriers and their categorization in the five sub-groups (Table 1), most of the subjects regarded individual factors to have an intermediate level importance among the barriers to family planning counseling (57.6% of the managers, 52.7% of the employees and 50.8% of the clients). Table 2 shows the frequency distribution of the responses to questions on individual barriers among the three groups of managers, employees and clients.

**Table 1.** Frequency Distribution of Viewpoint Scores in Three Groups of Managers, Employee and Clients in Relation with Individual Barriers to Family Planning Counseling

	Managers		Employees		Clients	
	Absolute Frequency	Relative Frequency	Absolute Frequency	Relative Frequency	Absolute Frequency	Relative Frequency
**Very little (0-20)**	0	0	4	3.6	6	4.8
**Little (21-40)**	9	15.3	21	19.3	46	36.5
**Intermediate (41-60)**	34	57.6	58	52.7	64	50.8
**Much (61-80)**	14	23.7	26	23.6	9	7.1
**Very much (81-100)**	2	3.4	1	0.9	1	0.8

**Table 2.** Relative Frequency Distributions (%) of the Responses to Viewpoint Questionnaire by the Managers, Employees and Clients

Individual Barrier	Absolutely Agree	Agree	No Idea	Disagree	Absolutely Disagree
**Clients' fear of the side effects of contraceptive method**					
Managers	23.7	64.4	8.5	3.4	0
Employees	26.4	65.5	0.9	4.5	2.7
Clients	20.6	62.7	5.6	9.5	1.6
**Clients' lack of confidence in the counselor or in the process of family planning counseling**					
Managers	3.4	25.4	25.4	45.8	0
Employees	2.7	28.2	23.6	35.5	10
Clients	3.2	12.7	15	53.2	15.9
**Insufficient or lack of updated personnel information about pregnancy prevention methods**					
Managers	3.4	15.3	3.3	66.1	11.9
Employees	0.9	14.5	10	57.3	17.3
Clients	1.6	10.3	19.1	61.1	7.9
**Not having a friendly manner by the counseler in providing the information related to the contraception issues to couples**					
Managers	3.4	33.9	10.1	45.8	6.8
Employees	2.7	30	7.3	48.2	11.8
Clients	1.6	4	17.4	63.5	13.5
**Having a sense of difficulty in envisioning the consultation process by the clients**					
Managers	5.1	23.7	33.9	35.6	1.7
Employees	2.7	30	26.3	35.5	5.5
Clients	4.8	24.6	4.7	55.6	10.3
**Clients' lack of interest in communicating and participating in family planning counseling**					
Managers	3.4	39	18.6	37.3	1.7
Employees	2.7	36.4	20	33.6	7.3
Clients	4	17.5	8.7	57.9	11.9
**Previous negative experiences of the clients in the process of family planning counseling**					
Managers	1.7	52.5	30.5	15.3	0
Employees	4.5	50	20.1	20.9	4.5
Clients	3.2	13.5	18.2	54	11.1
**Lack of adequate skills in the staff for counseling regarding the selection of contraceptive methods in special cases**					
Managers	10.2	40.7	20.3	28.8	0
Employees	3.6	39.1	17.3	30.9	9.1
Clients	7.9	30.2	11.2	44.4	6.3
**Too much tendency to use ineffective methods of contraception in the clients**					
Managers	30.5	54.2	11.9	3.4	0
Employees	30.9	52.7	7.3	6.4	2.7
Clients	12.7	25.4	10.3	34.9	16.7
**Discrimination of employees between the clients regarding the family planning counseling**					
Managers	3.4	10.2	23.7	45.8	16.9
Employees	0.9	6.4	15.5	50	27.2
Clients	2.4	18.3	19.8	46	13.5
**Employees' interest in one particular contraceptive method during family planning counseling**					
Managers	8.5	23.9	23.7	30.5	3.4
Employees	6.4	23.7	7.3	44.5	9.1
Clients	4.8	50.8	19	19.8	5.6

## 5. Discussion

Our results showed that the mean score of the perspectives of the managers, employees and clients about the individual barriers were different. On the other hand, most of the managers, employees and clients perceived these barriers as intermediate level barriers in the family planning counseling process. Therefore, strategic planning by the managers and policymakers of health services seems to be necessary to eliminate or modify individual barriers. Majlesi and colleagues (2011) and Glasier et al. (2008) found that the lack of knowledge about modern methods could cause fear in women (5, 8). In the present study, most of the clients, managers and employees agreed that clients had a major concern regarding the side effects of the selected contraceptive methods. This should be regarded as sign for the need for increasing the awareness of the clients about the methods of contraception and to address their concerns regarding the complications of contraceptive methods. Some of these concerns have been due to the norms, beliefs and their previous experiences; unfortunately, the staff ignored them and had a negative attitude towards these concerns. However, in an ideal service providing situation, employees should understand these concerns and address them properly. Therefore, enhancement of public awareness and particularly that of the authorities on the real goals of family planning and correction of misconceptions can lead to improvement in family planning services.

One of the most important problems in the healthcare providing system, is the lack of initiative knowledge and required skills in the employees (5-7, 9-11). In this study, most of the managers, employees and clients had opposing views regarding the lack of necessary and updated information about contraceptive methods among the staff. At the same time, most of managers, employees and clients had opposing views regarding the lack of adequate counseling skills in certain circumstances. On the other hand, during conducting this research, researchers repeatedly eyewitnesses that some of the staff did not have sufficient skills to help clients with certain conditions, which shows the need for follow-up and correction in this regard. Hence, it is necessary to run workshops and provide pamphlets that are more comprehensible. It also seems necessary to resolve this lack of professionalism. Baraitser et al. (2003) indicated that the staff's e attitude is an important barrier to access the family planning services (7). Meanwhile, Nalwadda et al. (2011) by interviewing with service providers and investigating the barriers for women in accessing the contraceptive methods, found that service providers who had false beliefs and attitudes regarding the process had a negative effect on receiving the proper contraception by young women (12). In the present study, most of managers and clients agreed and the most of the employees disagreed on the effect of the employees’ interest in a particular contraceptive method during counseling on family planning. On the other hand, the possible reason for clients’ agreeing on this issue could be due to the fact that they have been observing the same pattern of recommendations again and again across various counseling sessions. Therefore, it is necessary to take measures to solve this problem. Webber et al. (2012) found that there were contradictions between beliefs and attitudes of the family and that of the staff, which are considered a major obstacle in improving the reproductive health. Therefore, it is necessary to detect and modify these beliefs held by the family (13). In the present study, most of the subjects in all the three groups were disagreeing on the contribution of the employees' shyness and cultural barriers in preventing effective family planning counseling. However, Mohammad Alizadeh et al. (2008) have indicated employees' shyness in discussing family planning issues, particularly when speaking with men (9). Mohammad Alizadeh et al. (2009) and Kirimlioglu et al. (2005) in their studies about the family planning services have reported the silence and inactiveness of the clients in talking about their needs as a barrier in the consultation process (6, 14). In this study, most of managers and employees were agreeing on the clients’ lack of interest in participating in the process of family planning consultation. While most of the clients were disagreeing with this notion. It can be said that the clients’ unwillingness to participate could be due to the lack of participation opportunities offered by the staff. Mohammad Alizadeh et al. (2007) in a descriptive-observational study reported that two-thirds of service providers in their study did not encourage the clients to express their concerns and questions (15).

In this study most of the subjects opposed to the lack of trust of the clients in the counselors. Some previous studies such as Akers et al. (2007) have reported that the clients' trust in the counselors is an important factor in the cooperation of the clients with counselors (16). During their conversation with the researchers, some clients had expressed that they had uncertainty feelings during their first visit to the clinic. However, after some time, they were adapted to the environment and trusted in the healthcare employees. On the other hand, in the mentioned studies, the consultant was just acting as a consultant. However, in most cases, the family planning advisor was the same person who was providing also healthcare services for their children as well as family planning services. Artificially, in order to enhance their confidence level, most of the clients had the opposite tendency to mention the ineffective methods of contraception as a barrier to family planning counseling. Meanwhile, most of managers and employees did not agree with it, considering that the majority of the clients used condoms and the vast majority of clients used condoms after initially using the natural contraception methods. In a qualitative study, Carter et al. (2012) have reported that it was important for women to use contraceptive methods, but they feared the side effects and possibility of the failure of these methods. Despite having knowledge about the high failure rate of natural methods of contraception including the withdrawal method, this method was preferred as the method with fewer complications (17). It can be said that from the perspectives of women, having no side effects is more preferred than having a low failure rate. These findings showed the confidence of the clients in proper use of this method.

Bloom et al. (1999) argued that previous communications and encounters with health care professionals have created a sense of trust in the healthcare centers and encouraged women to use health services (18). In fact, communication experiences are a strong predictor of using reproductive health services by women. In this study, most of the managers and employees agreed that having negative experiences with previous consultants is a barrier for family planning counseling. However, most of the clients disagreed with this notion. It might be possible that clients' satisfaction can be more a result of the previous positive or negative experiences with various methods, for example the client might have had negative experiences of hormonal contraception and IUD methods which have common side effects. In Baraitser, Blake and Brown study (2003), it was implied that young clients who had participated in their study, viewed the family planning counseling as a cumbersome process and after passing it, they had the feeling of having had a big event (7). In our study, most of clients, managers and employees opposed to this notion as a barrier in family planning. It is possible that this difference with the results of Baraitser et al. study was due to the fact that subjects were chosen to be younger than 25 years of age in their study. Based on the above facts about the individual barriers, and the fact that a number of participants in each of the three groups agreed with these barriers, it is necessary to modify or eliminate them for enhancing the quality of family planning counseling through implementing new policies by the authorities. Meetings should be held with the presence of members of all the three groups of managers, employees and clients, in order to discuss the individual barriers and even other effective potential barriers in family planning counseling process. On the other hand, for improving the employees' performance, some measures should be taken such as professional training of health-care providers, especially in the faculties of nursing and midwifery regarding providing better health services, enhancing communication skills and also training physicians and managers in strategic planning. For effectively achieving better quality of the provided services, healthcare providers should receive targeted training with an emphasis on evidence-based resources such as recommendations of the World Health Organization in the field of quality improvement. 

## References

[1] Taghizade Z, Granmaie M, Vaseghrahimpour F (2009). Varny midwifery..

[2] Kohan SH, Taheri S, Fadai S, Baghersad Z (2013). comperhensive Textbook Of Maternal, Child& Reproductive Health..

[3] Kim MY, Lettenmaier C, Hopkins J (1999). Tools to Assess Family Planning Counseling:Observation and Interview..

[4] Farmand AA (2010). Quick Guide counseling individual based..

[5] Majlessi F, Moghaddam Banaem L, Shariat M (2011). Client and Health Workers Perceptions on Family Planning Services.. Iran Red Crescent Med J..

[6] Mohammad-Alizadeh S, Wahlstrom R, Vahidi R, Johansson A (2009). Women's perceptions of quality of family planning services in Tabriz, Iran.. Reprod Health Matters..

[7] Baraitser P, Blake G, Brown KC, Piper J (2003). Barriers to the involvement of clients in family planning service development: lessons learnt from experience.. J Fam Plann Reprod Health Care..

[8] Glasier A, Scorer J, Bigrigg A (2008). Attitudes of women in Scotland to contraception: a qualitative study to explore the acceptability of long-acting methods.. J Fam Plann Reprod Health Care..

[9] Mohammad-Alizadeh CS, Wahlstrom R, Vahidi R, Nikniaz A, Marions L, Johansson A (2009). Barriers to high-quality primary reproductive health services in an urban area of Iran: views of public health providers.. Midwifery..

[10] Mugisha JF, Reynolds H (2008). Provider perspectives on barriers to family planning quality in Uganda: a qualitative study.. J Fam Plann Reprod Health Care..

[11] Weston MR, Martins SL, Neustadt AB, Gilliam ML (2012). Factors influencing uptake of intrauterine devices among postpartum adolescents: a qualitative study.. Am J Obstet Gynecol..

[12] Nalwadda G, Mirembe F, Tumwesigye NM, Byamugisha J, Faxelid E (2011). Constraints and prospects for contraceptive service provision to young people in Uganda: providers' perspectives.. BMC Health Serv Res..

[13] Webber G, Spitzer D, Somrongthong R, Dat TC, Kounnavongsa S (2012). Facilitators and barriers to accessing reproductive health care for migrant beer promoters in Cambodia, Laos, Thailand and Vietnam: a mixed methods study.. Global Health..

[14] Kirimlioglu N, Elcioglu O, Yildiz Z (2005). Client participation and provider communication in family planning counselling and the sample study from Turkey.. Eur J Contracept Reprod Health Care..

[15] Mohammad-Alizadeh S, Marions L, Vahidi R, Nikniaz A, Johansson A, Wahlstrom R (2007). Quality of family planning services at primary care facilities in an urban area of East Azerbaijan, Iran.. Eur J Contracept Reprod Health Care..

[16] Akers AY, Gold MA, Borrero S, Santucci A, Schwarz EB (2010). Providers' perspectives on challenges to contraceptive counseling in primary care settings.. J Womens Health (Larchmt)..

[17] Carter MW, Bergdall AR, Henry-Moss D, Hatfield-Timajchy K, Hock-Long L (2012). A qualitative study of contraceptive understanding among young adults.. Contraception..

[18] Bloom SS, Lippeveld T, Wypij D (1999). Does antenatal care make a difference to safe delivery? A study in urban Uttar Pradesh, India.. Health Policy Plan..

